# Uncovering the phenotypic and transcriptional characteristics of biofilm-released cells in *Pseudomonas aeruginosa*

**DOI:** 10.3934/microbiol.2026031

**Published:** 2026-09-30

**Authors:** Jason Li, Larry Ayiku, Benjamin Larson, Agam Patel, Dylan Adams, Patrick Moran, Samantha Burgan, Esha Shah, Anthony Bruno, Jaclyn Printz, Tevin Flom, Whitni K. Redman

**Affiliations:** 1 Department of Biological Sciences, Binghamton University, Binghamton, NY, United States; 2 First-year Research Immersion Program, Binghamton University, Binghamton, NY, United States

**Keywords:** *Pseudomonas aeruginosa*, biofilm dispersion, biofilm-released cells, gene expression, antibiotic tolerance, bacterial attachment, biofilm reformation

## Abstract

*Pseudomonas aeruginosa* is a prominent biofilm-forming pathogen associated with chronic infections that are difficult to eradicate due to the protective biofilm matrix. Inducing biofilm dispersion has emerged as a potential therapeutic strategy. Although researchers have compared selected biofilm dispersion mechanisms, the characteristics of cells generated by mechanistically distinct release strategies, particularly enzymatic matrix degradation, remain uncharacterized. In this study, we compared *P*. *aeruginosa* cells released from biofilms using four distinct dispersion mechanisms: An external signaling molecule (*cis*-2-decenoic acid; *cis*-DA), a nutrient cue (glutamate), an environmental toxin (sodium nitroprusside; SNP), and a matrix-degrading enzyme (amylase). Released cells exhibited distinct antibiotic susceptibility, attachment, and gene expression depending on the release mechanism. Glutamate-released cells exhibited greater tobramycin tolerance and attachment, whereas glutamate and amylase-released populations exhibited differences in the expression of selected genes. Additionally, the release mechanism influenced subsequent biofilm recovery and reformation, and *cis*-DA significantly enhanced tobramycin-mediated killing of mature biofilms. Collectively, these findings demonstrate that the mechanism used to induce release may influence subsequent bacterial behavior and antibiotic susceptibility. These findings highlight the importance of considering release mechanism when developing dispersion-based strategies for treating biofilm-associated infections.

## 1. Introduction

Biofilms are communities of bacteria that are encased in an extracellular polymeric substance (EPS) [1]. Encapsulation within this matrix markedly reduces bacterial susceptibility to antimicrobial agents and host immune defenses [1]–[4]. The EPS also provides protection against exogenous stressors, limits phagocytosis by immune cells, and promotes enhanced surface colonization and long-term tolerance [2]–[4]. Collectively, these properties render biofilms a significant, clinical concern. The National Institutes of Health (NIH) estimates that close to 80% of all human infections involve bacteria that express a biofilm phenotype [5]. *Pseudomonas aeruginosa* is a Gram-negative, biofilm-associated pathogen, responsible for approximately 7.1 to 7.3% of all healthcare-associated infections [6]. *P. aeruginosa* biofilm-associated infections are often chronic and exceedingly difficult to eradicate due to elevated antibiotic tolerance conferred by the EPS [7]. Consequently, conventional treatment strategies frequently require prolonged, high-dose antibiotic regimens or invasive interventions such as surgical debridement [8]. The lack of efficient, non-invasive methods to clear biofilm infections underscores an urgent need for novel therapeutics capable of effectively disrupting biofilms and mitigating their clinical detriment.

A critical step toward developing improved anti-biofilm therapies is a thorough understanding of biofilm development and dispersal. Biofilm formation is a highly regulated, multistep process that has been broadly categorized into five stages: Reversible attachment, irreversible attachment, maturation 1, maturation 2, and dispersion [9],[10]. During the initial stages, planktonic cells attach to a surface and initiate biofilm development [9],[10]. Reversible attachment is characterized by weak, transient surface interactions during which cells may readily detach and return to their planktonic state [11]. Transition to irreversible attachment promotes cellular aggregation, quorum sensing induction, and microcolony formation [10],[11].

During maturation, these microcolonies expand and differentiate while producing increasing amounts of EPS, resulting in the complex three-dimensional architecture characteristic of mature biofilms [12]. The final stage of biofilm development, dispersion, has attracted considerable interest because it represents the stage during which cells exit the biofilm and disseminate to colonize new surfaces [13]. During dispersion, cells leave the biofilm matrix and transition to a temporary motile phenotype before adhering to nearby surfaces to initiate new biofilms. Because dispersed cells are no longer protected by the biofilm matrix, inducing biofilm dispersion has emerged as a promising strategy to improve antibiotic treatment of chronic biofilm-associated infections.

Dispersion has been broadly classified into two mechanisms: Active and passive. Active dispersion involves biologically regulated processes initiated by environmental cues or intracellular signaling pathways, whereas passive dispersion occurs through external physical or enzymatic disruption of the biofilm matrix [13]–[15]. Several experimental approaches have been developed to induce biofilm dispersion, including nutrient-based cues such as glutamate, signaling molecules such as *cis*-2-decenoic acid (*cis*-DA), nitric oxide donors such as sodium nitroprusside (SNP), and matrix-degrading enzymes such as amylase [16]–[20]. These approaches differ in their proposed mechanisms of action, with *cis*-DA and SNP primarily altering intracellular signaling, glutamate acting as a nutrient cue, and amylase disrupting extracellular matrix components [15]. Amylase was selected as the enzymatic release mechanism based on our previous systematic screening of 16 glycoside hydrolases targeting a range of polysaccharide linkages [19]. In that study, amylase from *A. oryzae* significantly dispersed *P. aeruginosa* biofilms *in vitro* and demonstrated dispersal activity in a more clinically relevant wound model. Additionally, amylase targets alpha-1,4 linkages, which are present in the *P. aeruginosa* exopolysaccharide Pel. Although several of these dispersion strategies have been investigated individually, relatively few researchers have directly compared the phenotypic consequences of these mechanistically distinct approaches under identical experimental conditions.

Researchers have directly compared selected mechanisms of *P. aerguinosa* biofilm dispersion and demonstrated that the mode of dispersion can influence the physiology of released cells. For example, actively dispersed cells generated by SNP and glutamate have been compared with spontaneously and passively dispersed populations, revealing differences in c-di-GMP levels, antibiotic susceptibility, motility, virulence-associated traits, and gene expression [16]–[18],[21]. These studies demonstrate that biofilm-released cells can differ from planktonic and biofilm-associated populations and that their physiological characteristics may depend on the mechanism of release. However, for comparative studies, researchers have primarily focused on signaling-mediated or physical mechanisms of dispersion, and comparatively less is known about how these populations compare with cells released through enzymatic degradation of the EPS. From a therapeutic perspective, biofilm dispersal is compelling because released cells often exhibit lower antibiotic tolerance than biofilm-associated bacteria following removal from the protective EPS matrix [18],[22],[23]. Nevertheless, enhanced virulence-associated traits or increased reattachment have also been reported following specific dispersion strategies, further suggesting that the biological consequences of induced biofilm release may depend on the mechanism employed [21],[24],[25]. Therefore, comparing signaling, nutrient, and matrix-degradation-mediated release within a common experimental framework may provide additional insight into how the mechanism of biofilm release influences the characteristics of released cells and the subsequent behavior of residual biofilms.

In this study, we systematically compared biofilm-associated cells released through four distinct dispersion mechanisms using *cis*-DA, glutamate, SNP, and amylase sourced from *Aspergillus oryzae* within a common experimental framework and evaluated biofilm release, antibiotic susceptibility, reversible attachment, expression selected genes, biofilm recovery, and biofilm reformation. To distinguish the effects of biofilm release from direct exposure to releasing agents, planktonic cells exposed to each treatment were also evaluated using growth curve analysis, phosphodiesterase activity assays, and RT-qPCR. Together, these experiments provide a direct comparison of phenotypic and transcriptional characteristics associated with four mechanistically distinct biofilm release strategies, and we evaluated how the method of biofilm release influences released-cell behavior and residual biofilm recovery.

## 2. Materials and methods

### 2.1. Bacterial strains

All experiments were performed using the organism *P. aeruginosa* PAO1 Parsek. Planktonic overnight cultures were grown in 125 mL flasks at 220 rpm at 37 °C for 16 h to 18 h in Lennox Broth (LB). To isolate cells in the exponential phase, a 1:100 diluted overnight was grown for either 3 h (full strength LB) or 5 h (1:5 LB) in 125 mL flasks at 220 rpm at 37 °C.

### 2.2. Preparation of Releasing agents

Releasing agent 310 nM *cis*-DA solution (Chemodex, Cat#DO249), 500 µM sodium nitroprusside (SNP) (SigmaAldrich, Cat#71778), and 18 mM glutamate (SigmaAldrich, Cat#G1251) solutions were prepared in assay-specific media as described previously [26]. A total of 500 U/mL α-amylase (from *Aspergillus oryzae*; SigmaAldrich, Cat#A9857) was prepared by dissolving lyophilized powder into the respective media and activated at 37 °C for 30 min prior to use [19],[20].

### 2.3. Growth curve

A total of 1:100 subcultures were grown for 12 h in 125 mL flasks shaking at 220 rpm at 37 °C. Experimental cultures were supplemented with the indicated releasing agents, whereas control cultures contained only LB. Optical density (OD600) was measured at 1 h intervals for 12 h. Three independent biological replicates were performed for each treatment.

### 2.4. *In vitro* phosphodiesterase (PDE) activity assay

Control cells were grown to exponential phase in LB, while experimental cells were grown to exponential phase in the presence of releasing agents. Cells were harvested by centrifugation (25,000 × g for 10 min), washed, and resuspended in PDE assay buffer (50 mM Tris, 50 mM NaCl, and 50 nM MgCl_2_, PH = 8). Cells were lysed by sonication and clarified by centrifugation. Total protein concentration was determined using the Pierce BCA Protein Assay (ThermoScientific, Cat# 23240). PDE activity was measured as an indirect indicator of c-di-GMP metabolism using the synthetic PDE substrate bis-(p-nitrophenyl) phosphate (bis-pNPP) substrate (TCI, Cat#B1099), as previously described [27],[28]. A total of 200 µg of total cell extract in PDE assay buffer was incubated with 100 mM bis-pNPP, and absorbance at 405 nm was measured every 30 min continuously for 20 h at room temperature. PDE activity was normalized based on total protein input rather than viable cell number (CFU) and was used as an indirect indicator of c-di-GMP metabolism, as hydrolysis of bis-pNPP provides a measure of phosphodiesterase activity associated with c-di-GMP degradation. This assay measures PDE activity rather than intracellular c-di-GMP concentrations directly.

### 2.5. *In vitro* well-plate biofilm models

Two biofilm models were employed to address different experimental objectives. The 2-day biofilm model was used primarily for downstream characterization of biofilm-released cells because the assays required relatively large numbers of viable released cells for subsequent standardization and analysis. Moreover, 2-day biofilms consistently provided a greater recoverable population of released cells suitable for phenotypic and molecular analyses including antibiotic susceptibility, reversible attachment, gene expression, biofilm recovery, and biofilm reformation. In contrast, mature 4-day biofilms were used for experiments designed to evaluate biofilm release and the efficacy of releasing agents in combination with antibiotic treatments. These established biofilms provided a robust mature biofilm model for assessing bacterial release and antibiotic-mediated killing in a more mature biofilm population. Importantly, the same biofilm age was used for all treatment groups within each experimental workflow, so comparisons among releasing agents within individual assays were performed under consistent biofilm growth conditions.

Following collection, all cell populations used for downstream phenotypic and molecular analyses were pelleted by centrifugation and the supernatant was carefully removed. Cell pellets were resuspended in 1 mL sterile saline to wash the cells and minimize carryover of releasing agents or other treatment components. Cells were then pelleted a second time, the saline wash was removed, and the washed cell pellets were resuspended in the appropriate medium for each downstream experiment.

### 2.6. *In vitro* well-plate biofilm model (4 days)

Biofilms were established in non-tissue culture-treated 24-well plates by inoculating 500 µL of 5 h subcultures grown in 1:5 LB into each well. Plates were incubated at 37 °C with shaking at 80 rpm for 4 days, with fresh media supplied every 12 h. Prior to treatment, wells were rinsed with 0.85% saline. Biofilms were treated with LB (control), releasing agents, 100 µg/mL tobramycin, or releasing agent + 100 µg/mL tobramycin for 2 h at 37 °C shaking at 80 rpm. Following treatment, supernatants containing released cells were collected separately from the remaining biofilm biomass. Residual biofilms were rinsed with 0.85% saline and collected into fresh saline by water sonication. Biofilm-associated cells and released cells were serially diluted and enumerated by drop-plating. Percentage released = CFU recovered from the supernatant/(CFU recovered from the supernatant + CFU recovered from the remaining biofilm) × 100.

### 2.7. *In vitro* well-plate biofilm model (2 days)

Biofilms were established by inoculating standardized subcultures (10^6^ CFU/mL) into non-tissue culture-treated 24-well plates and incubating for 48 h at 37 °C shaking at 80 rpm. Following incubation, unattached cells were removed by rinsing with 0.85% saline. Biofilms were treated with LB (control) or the indicated releasing agents for 2 h. Released cells were collected for downstream analyses.

### 2.8. Microscopy of released cells

A total of 10 µL of released cells obtained from 2-day biofilms were mounted to a glass microscope slide (Globe Diagnostics) and heat-fixed. The slides were stained with Advanced Counterstain (Hardy Diagnostics) for 1 min. Excess stain was rinsed with deionized water. The slides were dried with bibulous paper for 10 min, wet-mounted with immersion oil, and imaged under oil immersion with 100× magnification using brightfield microscopy (CellSens Entry software and Olympus microscope camera). Microscopy was performed for qualitative assessment of released-cell morphology and aggregation. Representative images are presented for visualization only and were not subjected to quantitative image analysis.

### 2.9. Released cell susceptibility

Released cells were collected from the 2-day biofilms, centrifuged at 16,000 × g for 2 min, and resuspended in fresh LB. The suspended cells were standardized to an OD600 of 0.2. Cell suspensions were treated with either LB or 50 µg/mL of tobramycin for 1 h at 37 °C with shaking at 220 rpm. Post-treatment, cells were serially diluted and drop-plated to enumerate CFU/mL. Exponential-phase planktonic cells, stationary-phase planktonic cells, and biofilm-associated cells were included as reference controls.

### 2.10. Released cell attachment assay

Released cells were collected from 2-day biofilms, standardized to an OD600 of 0.2, and inoculated into 96-well plates for 2 h at 37 °C shaking at 220 rpm. Following incubation, unattached cells were removed, and attached cells were stained with 0.1% crystal violet for 10 min. Excess stain was rinsed off with distilled water, and plates were dried at room temperature overnight. The stained cells were then eluted with 95% ethanol for 15 min and read at an absorbance at 570 nm.

Exponential-phase planktonic cells, stationary-phase planktonic cells, and biofilm-associated cells were included as reference controls.

### 2.11. RNA extraction

Biofilm-released cells, residual biofilm cells, stationary-phase cells, and exponential-phase cells were collected from 2-day biofilms and washed as described above. Cells were stabilized in RNAlater and stored at −80 °C prior to RNA extraction using the E.Z.N.A.(R) Bacterial RNA Kit (Omega Bio-Tek, Cat#R6950-01) according to the manufacturer's guidelines. DNase treatment was performed using the E.Z.N.A(R) RNase– Free DNase 1 Set (Omega Bio-Tek, CatE1091) according to the manufacturer's instructions.

### 2.12. cDNA synthesis8

Total RNA (100 ng) was reverse transcribed using qScript Ultra SuperMix(R) reaction kit (QuantaBio, Cat#95217) for a final reaction volume of 20 µL. The reaction mixture was incubated at 25 °C for 2 min to anneal the primers, 55 °C for 10 min to reverse transcribe the RNA, and enzyme was inactivated at 95 °C for 1 min. cDNA was stored long-term at −80 °C.

### 2.13. RT-qPCR

Gene-specific primers in Table S1 were used for amplification using PerfeCTa(R) SYBR(R) Green FastMix(R) (Quantabio, Cat#95072) on a Bio-Rad CFX96 Real-Time PCR Detection System. Expression of each target gene was normalized to the housekeeping gene *rho*, encoding the transcription termination factor Rho. A single reference gene was used for these analyses, and the stability of *rho* was not independently evaluated across the individual conditions. Relative gene expression was calculated using the comparative ΔΔC_T_ method. For biofilm-released cells, relative expressions were normalized to LB-released cells or untreated exponential-phase planktonic cells (Figure S1). Each RT-qPCR experiment included 2–3 independent biological replicates, with each biological replicate analyzed in technical duplicate. Values from technical duplicates were averaged, so each biological replicate contributed to a single value to the statistical analysis.

### 2.14. Biofilm recovery and reformation

Washed cell populations collected as described above were evaluated for biofilm recovery and reformation. Residual biofilms were replenished with fresh LB to evaluate recovery of the original biofilm. Biofilms were incubated for 24, 48, or 72 h with media replacement every 24 h. Biofilm biomass was quantified using crystal violet staining followed by ethanol solubilization and absorbance measurement at 570 nm.

### 2.15. Statistical analysis

All statistical analysis was performed utilizing GraphPad Software, Inc. Normality and homogeneity of variance were evaluated prior to statistical analysis. Growth curve data were analyzed using two-way repeated-measures ANOVA followed by Dunnett's multiple-comparisons test. When assumptions of equal variance were not met, Brown-Forsythe and Welch one-way ANOVA followed by Games-Howell or Dunnett's multiple comparisons tests were performed. Antibiotic susceptibility and attachment data were analyzed using Brown-Forsythe and Welch ANOVA followed by Dunnett's T3 multiple-comparisons test with multiplicity-adjusted *p* values. Gene expression data were analyzed using Brown-Forsythe and Welch ANOVA followed by Games-Howell multiple-comparisons testing. Multiplicity-adjusted *p* values were used for pairwise comparisons. For interpretation of RT-qPCR results, statistical significance and fold-change magnitude were considered. Expression differences ≥ 2-fold with an adjusted *p* < 0.05 were highlighted as changes of potential biological relevance. Biofilm killing experiments were analyzed using two-way ANOVA followed by Sidak's multiple-comparisons test. Statistical tests used for individual experiments are indicated in the corresponding figure legends. Each data point shown in the figures represents one independent biological replicate. The number of biological replicates performed for each experiment is reported in the corresponding figure legend.

## 3. Results

### 3.1. Releasing agents produce minimal physiological changes in planktonic cells

To distinguish the direct effects of releasing agents on planktonic cells from phenotypic changes associated with biofilm release, planktonic *P. aeruginosa* cultures were exposed to each releasing agent prior to evaluating bacterial growth and phosphodiesterase (PDE) activity. Growth curves were performed to determine whether the releasing agents altered bacterial growth, while PDE activity was measured as an indirect indicator of intracellular cyclic di-GMP (c-di-GMP) metabolism.

*P. aeruginosa* subcultures were grown for 12 h in LB alone or LB supplemented with a releasing agent. Optical density at 600 nm was measured hourly to monitor bacterial growth (Figure 1A). No significant differences in bacterial growth were observed for glutamate or amylase-exposed cultures compared to the LB control, indicating that these releasing agents did not measurably affect bacterial growth. In contrast, SNP exposure resulted in slight reductions in growth at 4 h and 5 h, but these differences were not statistically significant, and growth recovered to control levels by 6 h. *Cis*-DA exposure produced a modest but statistically significant reduction of bacterial growth at 7 h (*p* = 0.0411), with no significant differences observed at the remaining time points. These findings indicate that, under the conditions tested, the releasing agents produced little to no sustained effect on planktonic bacterial growth.

To determine whether exposure to releasing agents altered intracellular c-di-GMP levels, PDE activity was measured in exponential-phase planktonic cultures exposed to each releasing agent and compared with untreated exponential-phase and stationary-phase controls (Figure 1B). SNP-exposed cultures exhibited the highest mean PDE activity (0.386 nM/min/mg), whereas *cis*-DA exposed cultures exhibited the lowest mean PDE activity (0.044 nM/min/mg). However, none of the releasing agent-treated groups differed significantly from the untreated exponential-phase control.

When compared with stationary-phase cells, exponential-phase cells (*p* = 0.0309), SNP-exposed cultures (*p* = 0.0234), and glutamate-exposed cultures (*p* = 0.0016) exhibited significantly greater PDE activity, consistent with increased c-di-GMP degradation and potentially reduced intracellular c-di-GMP levels. These comparisons provide context for differences between planktonic growth phases but should not be interpreted as evidence that exposure to the releasing agents significantly altered PDE activity relative to untreated exponential-phase cells. Collectively, these results indicate that exposure to the releasing agents had minimal direct effects on planktonic bacterial growth and PDE activity under the conditions tested. Consequently, the phenotypic and transcriptional differences observed in subsequent experiments are more likely to reflect characteristics of biofilm-released cells rather than direct physiological responses to the releasing agents alone.

### 3.2. Releasing agents promote cell release from mature 4-day biofilms

To determine the ability of the four releasing agents to promote biofilm cell release, mature 4-day *P. aeruginosa* biofilms were treated for 2 h with *cis*-DA, glutamate, SNP, amylase, or LB control. Following treatment, released cells in the supernatant and cells remaining within the biofilm were collected and quantified via drop-plating to enumerate CFU/mL to calculate the percentage of cells released.

All four releasing agents significantly increased cell release compared to the LB control. Untreated biofilms released 28.7% of the total bacterial population, whereas glutamate, SNP, cis-DA, and amylase released 77.5%, 95.4%, 87.9%, and 96.6%, respectively. Among the treatments, glutamate produced the lowest percentage of released cells but remained significantly greater than the LB control (p = 0.0008). SNP and amylase exhibited the greatest release of cells, while *cis*-DA had an intermediate level of release (Figure 2). Differences in the morphology of released populations were also observed. SNP and *cis*-DA primarily released individual bacterial cells, whereas amylase treatment released multicellular aggregates that remained partially associated with the biofilm matrix (Figure S1). The phenotypic consequences of these differences were investigated in subsequent experiments. All four releasing agents induced measurable release of cells from mature 4-day biofilms. Although the percentage of released cells varied numerically among treatments, no statistically significant differences in the extent of release were detected among the four releasing agents.

**Figure 1.**
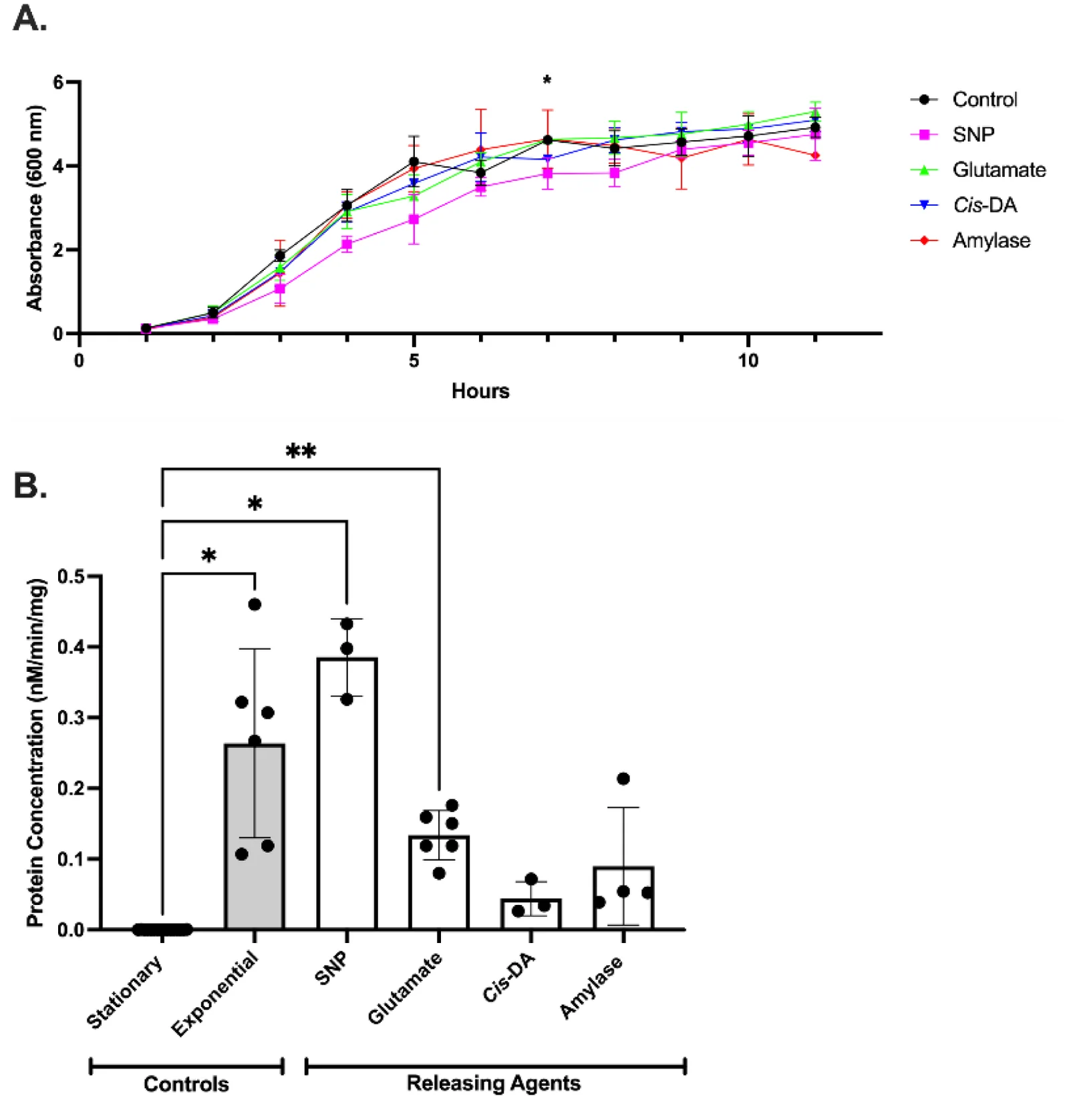
Direct effects of releasing agents on planktonic *Pseudomonas aeruginosa* cells. Planktonic PAO1 cultures were exposed to LB (control) *cis*-DA, glutamate, SNP, or amylase to evaluate whether the releasing agents directly affected bacterial physiology. (A) Bacterial growth was monitored by measuring optical density at 600 nm every hour for 12 h. Statistical significance at each time point was determined using two-way repeated-measures ANOVA with Dunnett's multiple comparison test, with each treatment compared to the LB control. (B) PDE activity was measured in exponential-phase planktonic cultures following exposure to each releasing agent. Untreated exponential-phase and stationary-phase cultures served as controls. PDE activity was measured using 200 µg of total cell protein. Statistical significance was determined using Brown-Forsythe and Welch one-way ANOVA followed by Games-Howell multiple comparisons test compared to stationary phase (black) or exponential phase (grey) controls. Mean ± standard deviation is shown. (*p < 0.05; **p < 0.01; ***p < 0.001). n = 3/group for growth curve and n = 3–16 for PDE activity.

**Figure 2.**
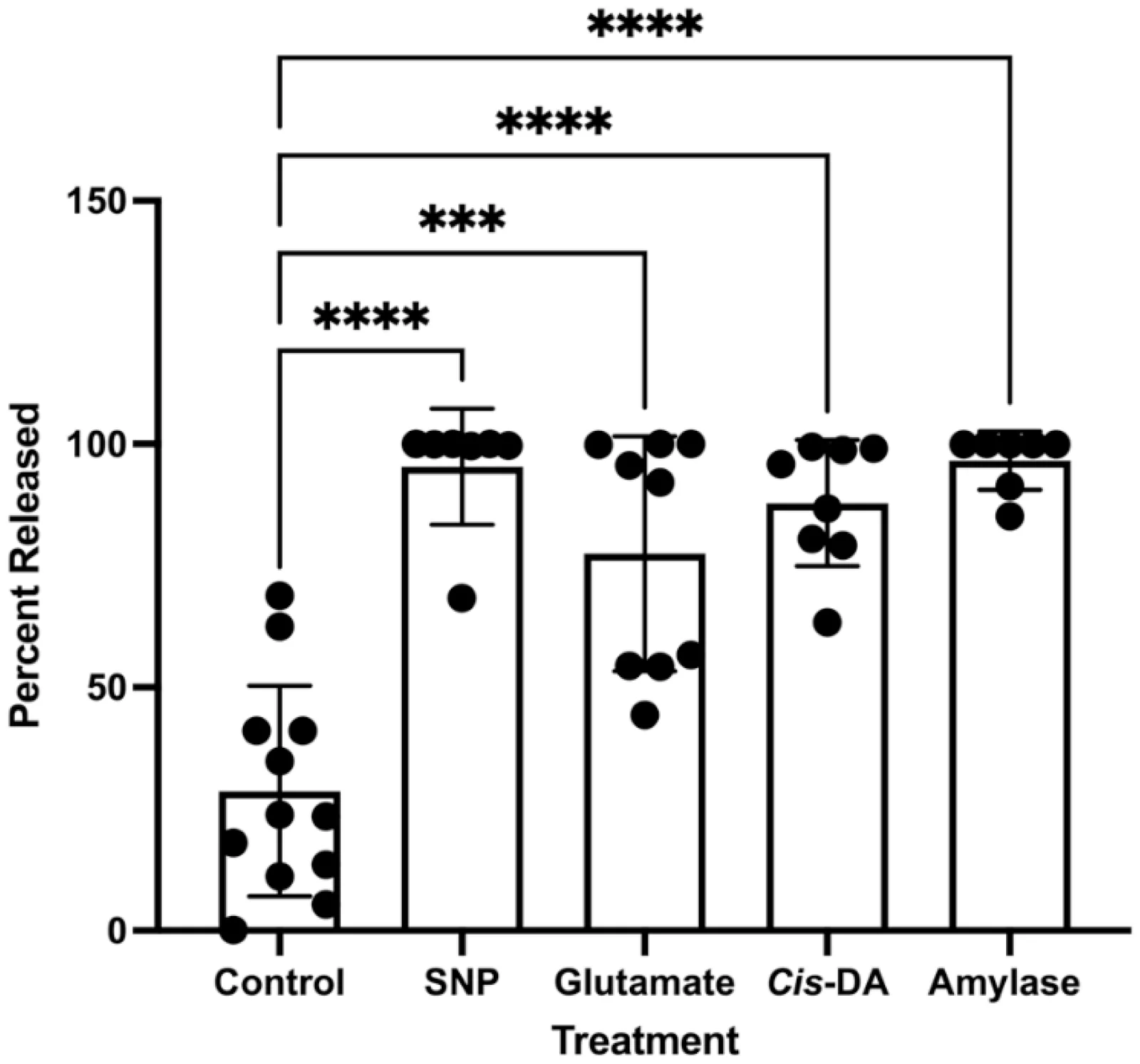
Releasing agents significantly increase the release of cells from mature 4-day *P. aeruginosa* biofilms. Mature 4-day PAO1 biofilms were treated for 2 h with LB (control), *cis*-DA, glutamate, SNP, or amylase. Following treatment, released cells in the supernatant and cells remaining within the biofilm were collected, serially diluted, and drop-plated to enumerate CFU/mL. The percentage of released cells was as (CFU supernatant/total CFU) x 100. Statistical significance was determined using Brown-Forsythe and Welch one-way ANOVA followed by Dunnett's multiple comparisons test with each treatment compared to the LB control. Mean ± standard deviation is shown (*p < 0.05; **p < 0.01; ***p < 0.001; ****p < 0.0001). n = 7–12/group.

### 3.3. Glutamate-released cells exhibit the greatest tolerance to tobramycin

Released cells collected from 2-day *P. aeruginosa* biofilms were evaluated to assess their tolerance to tobramycin. Briefly, released cells were treated with 50 µg/mL tobramycin for 1 h, after which samples were serially diluted and drop-plated to determine CFU/mL. Antibiotic tolerance was assessed by calculating the log reduction in viable cells relative to untreated controls and comparing released-cell populations with exponential-phase, stationary-phase, and biofilm-associated cells.

As expected, biofilm-associated cells exhibited the smallest log reduction (1.9 log_10_ CFU/mL), consistent with their greater tolerance to tobramycin compared with planktonic controls (Figure 3). Among the released populations, SNP-, *cis*-DA-, and amylase-released cells exhibited similar log reduction with 1.87, 2.47, and 2.13 log_10_ CFU/mL, respectively. In contrast, glutamate-released cells exhibited the smallest log reduction (1.15 log_10_ CFU/mL) among the released populations and demonstrated significantly greater tolerance than exponential-phase cells (*p* = 0.0084). No significant differences were observed between glutamate-released cells and biofilm-associated cells. These findings indicate that the mechanism used to release biofilm cells influenced their subsequent response to tobramycin. Pairwise comparisons demonstrated that glutamate-released cells differed significantly from exponential planktonic cells (*p* = 0.0084), whereas SNP (*p* = 0.0818), *cis*-DA (*p* = 0.7557), and amylase-released cells (*p* = 0.2056) were not significantly different from exponential planktonic cells. These findings indicate that the antibiotic susceptibility of biofilm-released cells varies depending on the mechanism of release.

**Figure 3.**
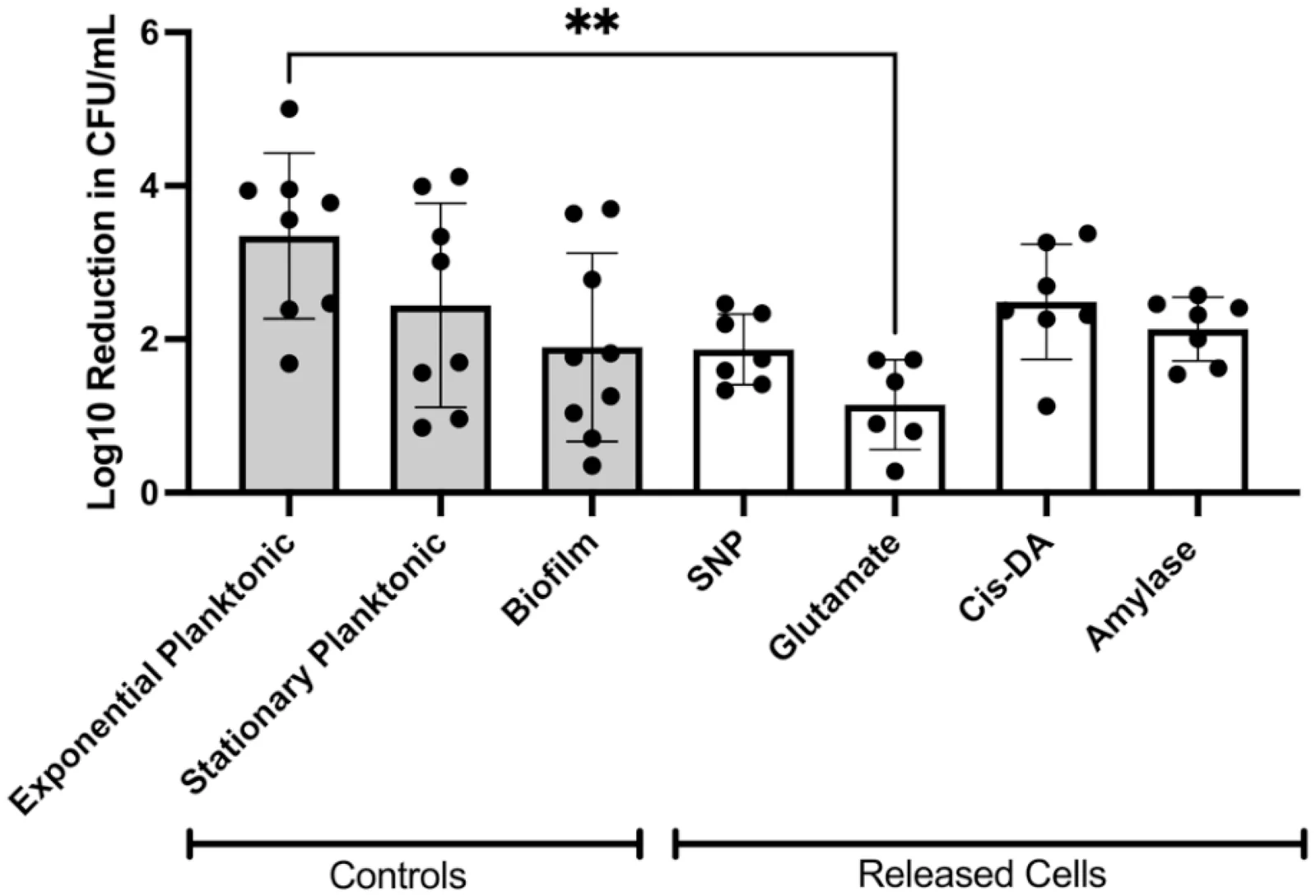
Antibiotic tolerance of biofilm-released *Pseudomonas aeruginosa* cells following treatment with four releasing agents. Released cells obtained from 2-day PAO1 biofilms were treated with *cis*-DA, glutamate, SNP, or amylase. Released cells, exponential-phase cells, stationary-phase cells, and biofilm-associated cells were adjusted to an OD600 of 0.2 and exposed to either LB (media control) or 50 µg/mL tobramycin for 1 h. Following treatment, samples were serially diluted and drop-plated to calculate log reduction compared to the LB treated control. Statistical significance was determined using Brown-Forsythe and Welch one-way ANOVA followed by Dunnett's T3 multiple comparisons test with adjusted *p* values to compare each treatment to the exponential, stationary, and biofilm controls. Mean ± standard deviation is shown (*p < 0.05; **p < 0.01). n = 6–9/group.

### 3.4. Released cells exhibit distinct reversible attachment profiles

To evaluate the reversible attachment of released cells generated by different release mechanisms, 2-day *P. aeruginosa* biofilms were treated with each releasing agent, and released cells were collected, pelleted, and resuspended in fresh LB medium. Cell suspensions were incubated in 96-well plates for 2 h to enable reversible attachment. Attached cells were quantified using crystal violet and absorbance measurements at 570 nm. Exponential-phase cells, stationary-phase cells, and biofilm-associated cells were included as reference controls.

Released-cell populations exhibited distinct attachment behaviors depending on the release mechanism (Figure 4). SNP and *cis*-DA released cells exhibited the lowest levels of reversible attachment among the released populations, with absorbance values of 0.86 and 0.91, respectively, similar to stationary-phase cells (0.82). All released-cell populations differed significantly from exponential-phase cells. In contrast, glutamate-released cells exhibited greater reversible attachment (1.52), approaching the level observed for biofilm-associated cells (1.75). Pairwise comparisons among released-cell populations demonstrated that glutamate-released cells exhibited significantly greater attachment than SNP (*p* = 0.0039) and *cis*-DA released cells (*p* = 0.0059). Similarly, amylase-released cells exhibited significantly greater attachment than SNP (*p* = 0.0048) and *cis*-DA released cells (*p* = 0.0074). No significant difference was observed between glutamate and amylase released cells (*p* = 0.9987) or between SNP and cis-DA released cells (*p* > 0.999). Amylase released cells also exhibited significantly greater attachment than stationary-phase cells (*p* = 0.0015). These findings demonstrate that the mechanism used to release biofilm cells influence their subsequent attachment behavior. SNP and *cis*-DA-released cells exhibited lower reversible attachment, whereas glutamate and amylase-released cells retained greater attachment capacity under the conditions examined.

**Figure 4.**
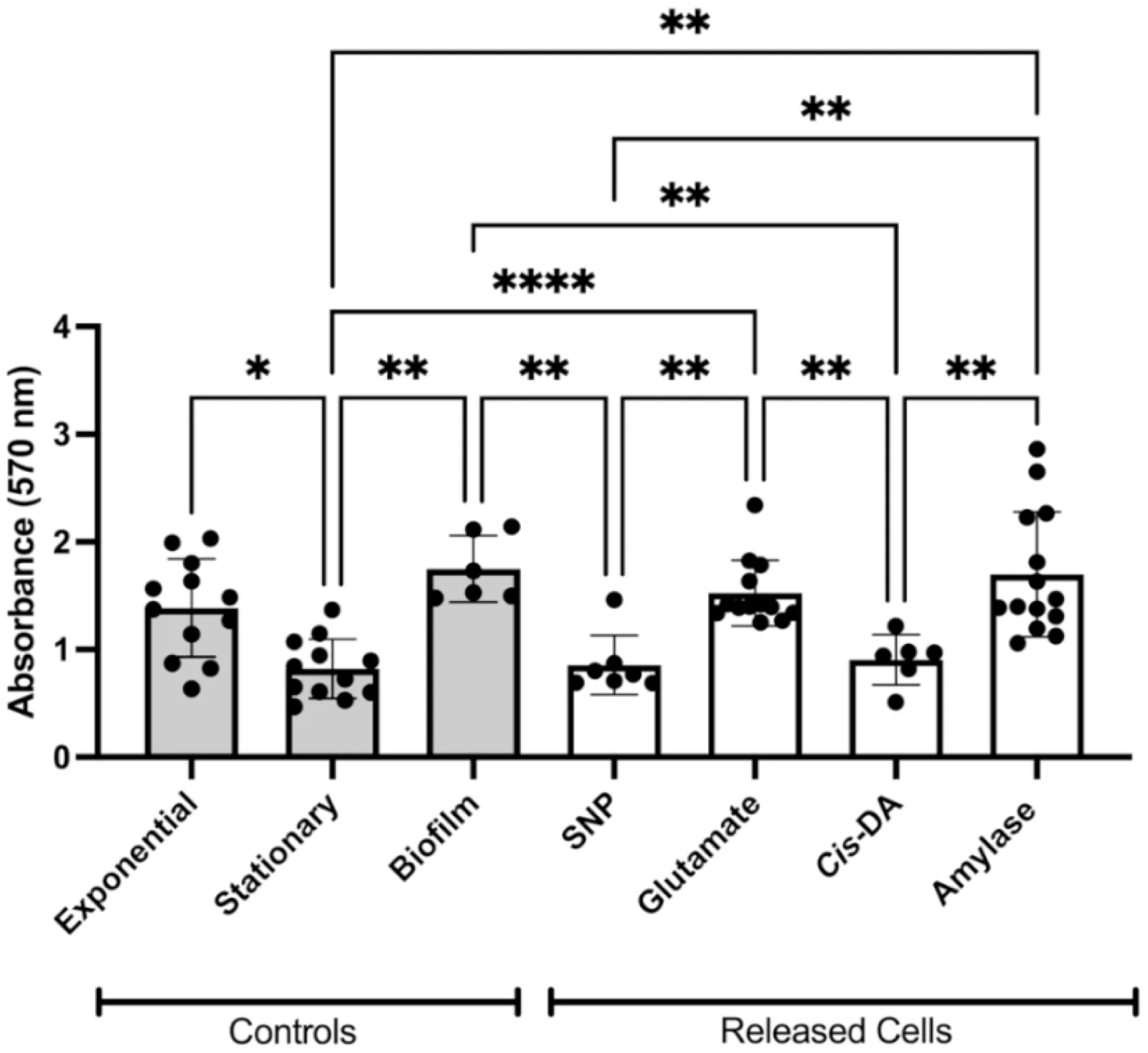
Reversible attachment of biofilm-released *P. aeruginosa* cells following treatment with four releasing agents. 2-day PAO1 biofilms were treated with *cis*-DA, glutamate, SNP, or amylase. Released cells, exponential-phase cells, stationary-phase cells, and biofilm-associated cells were adjusted to an OD600 of 0.2 and inoculated in 96-well plates for 2 h to assess reversible attachment. Attached cells were stained with crystal violet and retained biomass was quantified by measuring absorbance at 570 nm following elution in ethanol. Statistical significance was determined via Brown-Forsythe and Welch one-way ANOVA followed by Dunnett's T3 multiple comparison test to compare each treatment to the exponential, stationary, and biofilm controls. Mean ± standard deviation is shown (*p < 0.05; **p < 0.01; ***p < 0.001; ****p < 0.0001). n = 6–14/group.

### 3.5. Releasing agents are associated with differences in gene expression of biofilm-released cells

To determine whether the mechanism of biofilm release influenced bacterial gene expression, RT-qPCR was performed on biofilm-released cells following treatment with SNP, glutamate, *cis*-DA, or amylase from 2-day biofilms. Statistical significance and fold-change magnitude were considered when interpreting transcriptional differences, with a ≥ 2-fold change and adjusted *p* < 0.05 used to highlight changes of potential biological relevance. Gene expression was evaluated for genes associated with motility (*fliC* and *pilA*), biofilm formation (*cdrA*), dispersion (*bdlA*), antibiotic resistance (*mexR*), quorum sensing (*lasR*), and the acute-to-chronic transition (*sicX*). Relative gene expression of released cells was normalized to LB-released cells to identify transcriptional differences associated with each release mechanism (Figure 5). Gene expression normalized to exponential-phase planktonic cells is provided as Figure S1 to facilitate comparison with planktonic populations.

Among the motility-associated genes, *fliC* expression was similar across all released-cell populations, whereas *pilA* expression differed among release mechanisms (Figure 5A,B). SNP-released cells (fold change = 1.245) exhibited significantly greater *pilA* expression than glutamate-released cells (fold change = −1.32) (*p* = 0.0027) and amylase-released cells (fold change = −1.63; *p* = 0.047), while no other pairwise differences were detected. Although this difference reached statistical significance, the magnitude of the change was <2-fold and was therefore interpreted cautiously as a small transcriptional change. Expression of genes associated with biofilm regulation exhibited greater variability between release mechanisms. Although *cdrA* expression tended to be higher in amylase-released cells (fold change = 9.86), no statistically significant differences were observed among treatments (Figure 5C). In contrast, *bdlA* expression differed among released-cell populations, with glutamate-released cells (fold change = 17.62) exhibiting greater expression than SNP-released cells (fold change = −3.25), while amylase-released cells (fold change = 9.76) also exhibited significantly greater *bdlA* expression than SNP-released cells (*p* = 0.047) (Figure 5D). Although glutamate exhibited a numerically greater mean fold change than amylase, the glutamate comparison did not reach statistical significance, reflecting greater variability among biological replicates.

Similarly, expression of the multidrug resistance regulator *mexR* varied according to the release mechanism (Figure 5E). Amylase-released cells (fold change = 3.91) exhibited significantly greater *mexR* expression than SNP-released cells (fold change = −2.64; *p* = 0.0263), whereas glutamate-released cells (fold change = 1.78) exhibited significantly greater expression than *cis*-DA-released cells (fold change = −1.6; *p* = 0.0007). Expression of *lasR* also differed among released-cell populations (Figure 5F). SNP-released cells (fold change = −1.92) exhibited significantly lower *lasR* expression than *cis*-DA-released cells (fold change = −1.22; *p* = 0.0357). In contrast, *sicX* expression did not differ significantly among treatments (Figure 5G).

Normalization to exponential-phase planktonic cells produced similar overall trends (Figure S1). Notably, glutamate and amylase-released cells (fold changes = 55.04 and 31.5, respectively) exhibited the greatest increases in *bdlA* expression relative to exponential-phase cells, while amylase-released cells also exhibited the highest *cdrA* and *mexR* expression (fold change = 19.5 and 16.85, respectively). These findings support the observation that glutamate and amylase-mediated release produced the largest transcriptional shifts among the released-cell populations.

Collectively, these findings provide preliminary evidence that the mechanism used to release biofilm cells may influence the expression of selected regulatory genes. However, not all statistically significant comparisons exhibited large fold changes, and the limited number of biological replicates warrants cautious interpretation of these transcriptional differences. Given the limited number of biological replicates, these transcriptional differences should be interpreted cautiously and require validation with larger independent datasets.

**Figure 5.**
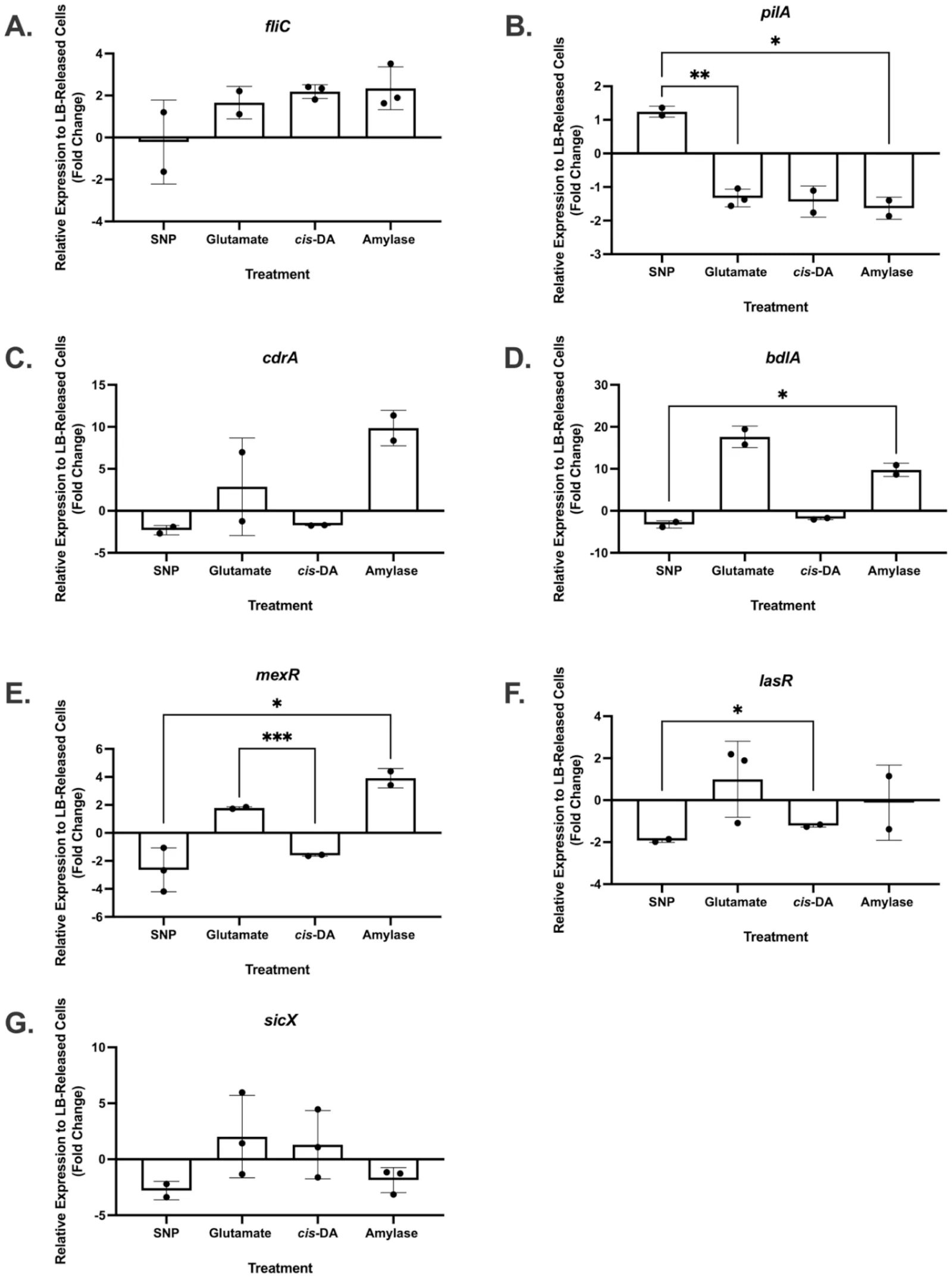
Gene expression of biofilm-released *Pseudomonas aeruginosa* cells normalized to LB-released control. RT-qPCR analysis of gene expression in PAO1 cells released from 2-day biofilms following treatment with LB (control), SNP, glutamate, *cis*-DA, or amylase. Released cells were collected, RNA was extracted, and relative gene expression was calculated using the ΔΔCt method normalized to cells released following LB treatment. Panels represent relative gene expression for (A) *fliC*, (B) *pilA*, (C) *cdrA*, (D) *bdlA*, (E) *mexR*, (F) *lasR*, and (G) *sicX*. Statistical significance was determined using Brown-Forsythe and Welch ANOVA followed by Games-Howell multiple-comparisons testing with multiplicity-adjusted *p* values. Mean ± standard deviation is shown (*p < 0.05; **p < 0.01). n = 2–3 independent biological replicates/group, with each biological replicate analyzed in technical duplicate and technical duplicate values averaged prior to statistical analysis.

### 3.6. Biofilm recovery and biofilm reformation differ according to the release mechanism

To determine whether established biofilms recovered following treatment and whether released cells retained the ability to form new biofilms, 2-day *P. aeruginosa* biofilms were treated with LB (control) or one of the four releasing agents. Released cells were collected, centrifuged, and resuspended in fresh LB prior to inoculation into new 24-well plates to assess biofilm reformation. The remaining biofilm biomass was replenished with fresh LB and incubated to evaluate recovery of the original biofilm. Biofilm biomass was quantified after 24, 48, or 72 h using crystal violet staining (Figure 6). Crystal violet staining reflects total surface-associated biomass rather than viable bacterial numbers, and measurements at each time point were obtained from independently sampled biofilms. Therefore, changes in absorbance across time points were interpreted as differences in retained biofilm biomass rather than continuous increases or decreases in bacterial growth.

Following 24 h, significant differences in surface-associated biomass were observed among treatments (Figure 6A). SNP and amylase-treated biofilms exhibited significantly greater biomass than LB-treated controls (absorbance = 4.21, 3.58; *p* = 0.0069, 0.0016, respectively), whereas *cis*-DA-treated biofilms exhibited less biomass relative to all other treatments (absorbance = 2.14). By 48 h, no significant differences in recovering biofilm biomass were observed among treatments (Figure 6C). At 72 h, significant differences among treatment groups were again observed (Figure 6E). SNP-treated biofilms exhibited significantly lower biomass than LB-treated controls (absorbance = 1.28; *p* = 0.0012), whereas *cis*-DA- and amylase-treated biofilms demonstrated significantly greater biomass than SNP-treated biofilms (absorbance = 3.91 and 4.61 vs 1.28, respectively). Thus, significant differences among treatment groups were observed at 24 and 72 h but not at 48 h. Because these differences were not consistently observed across all time points, they were interpreted as time-specific differences in surface-associated biomass rather than evidence of a consistent pattern in biofilm recovery.

Biofilm formation by released cells also differed among treatments (Figure 6B,D,F). After 24 h, no significant differences in biofilm biomass were observed among the released-cell populations (Figure 6B). At 48 h, amylase-released cells formed significantly greater biofilm biomass than SNP-released cells (absorbance = 5.06 vs 3.74; *p* = 0.0073), while the remaining treatments exhibited intermediate biomass that did not differ significantly (Figure 6D). Similarly, after 72 h, *cis*-DA- and amylase-released cells formed significantly greater biofilm biomass than SNP-released cells (absorbance = 3.47 and 3.59 vs 1.08, respectively) (Figure 6F). No significant differences were detected among the remaining treatment groups.

Collectively, these findings demonstrate time-specific variation in surface-associated biomass during recovery of treated biofilms and biofilm formation by released cells. However, differences among treatments were not consistently observed at all time points, and some statistically significant differences represented relatively small changes in crystal violet staining. Therefore, these findings should be interpreted as differences in surface-associated biofilm biomass under the conditions examined rather than corresponding changes in bacterial viability.

**Figure 6.**
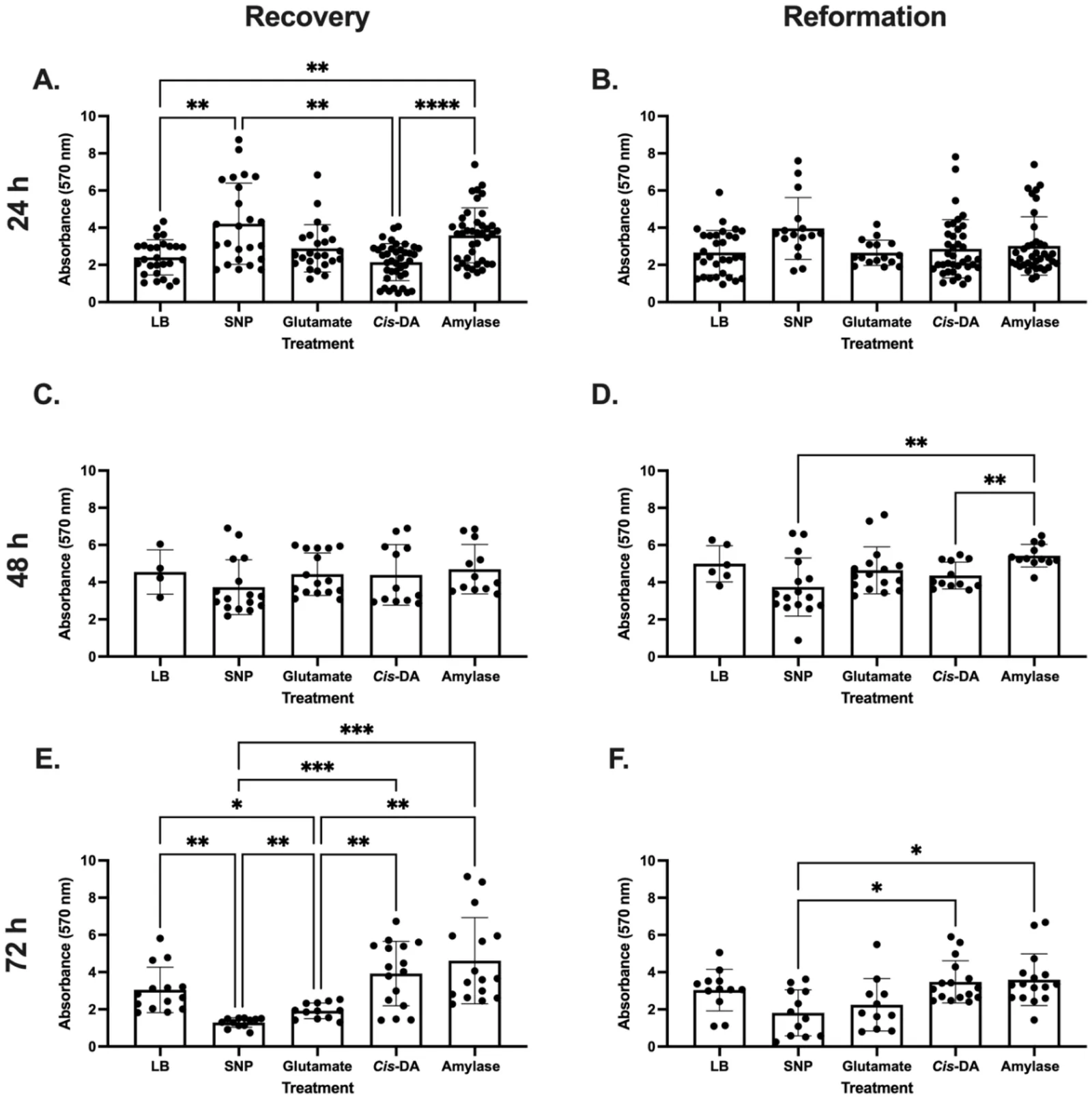
Recovery of treated biofilms and biofilm formation by released *Pseudomonas aeruginosa* cells. 2-day PAO1 biofilms were treated with LB (control), SNP, glutamate, *cis*-DA, or amylase. Released cells were collected, centrifuged, and resuspended in fresh LB, and inoculated into new 24-well plates to assess biofilm formation. The remaining treated biofilms were replenished with fresh LB to evaluate recovery of the original biofilm. Recovering biofilms (A, C, E) and biofilms formed by released cells (B, D, F) were incubated for 24 (A, B), 48 (C, D), or 72 h (E, F). Following incubation, biofilms were stained with crystal violet, rinsed with distilled water, and eluted in ethanol. The absorbance was measured at 570 nm. Statistical significance was determined via Brown-Forsythe and Welch one-way ANOVA followed by Dunnett's multiple comparisons test. Mean ± standard deviation is shown (*p < 0.05; **p < 0.01; ***p < 0.001; ****p < 0.0001). n = 4–40/group.

### 3.7. Releasing mechanisms differentially influence tobramycin-mediated biofilm killing

To determine whether biofilm release influenced antibiotic-mediated killing, 4-day *P. aeruginosa* biofilms were treated for 2 h with LB (control), 100 µg/mL tobramycin alone, or each releasing agent with or without 100 µg/mL tobramycin. Following treatment, the remaining biofilm biomass was rinsed to remove residual antibiotic, collected, homogenized, serially diluted, and drop-plated to enumerate CFU/mL (Figure 7).

Among the releasing-agent and antibiotic combinations evaluated, *cis*-DA combined with tobramycin (2.9 × 10^4^ CFU/mL) exhibited a significant reduction in viable biofilm cells compared with *cis*-DA treatment alone (1.3 × 10^7^ CFU/mL; *p* = 0.0015). Although viable cell counts following SNP and amylase in combination with tobramycin were numerically reduced below the clinically relevant threshold of 10⁵ CFU/mL [19],[29],[30], these reductions did not reach statistical significance. This clinically derived value was provided only as a reference point and was not been established as a threshold for therapeutic efficacy in the present *in vitro* biofilm model. Therefore, while these findings may be clinically relevant, the effects of SNP and amylase should be interpreted cautiously and require further investigation.

**Figure 7.**
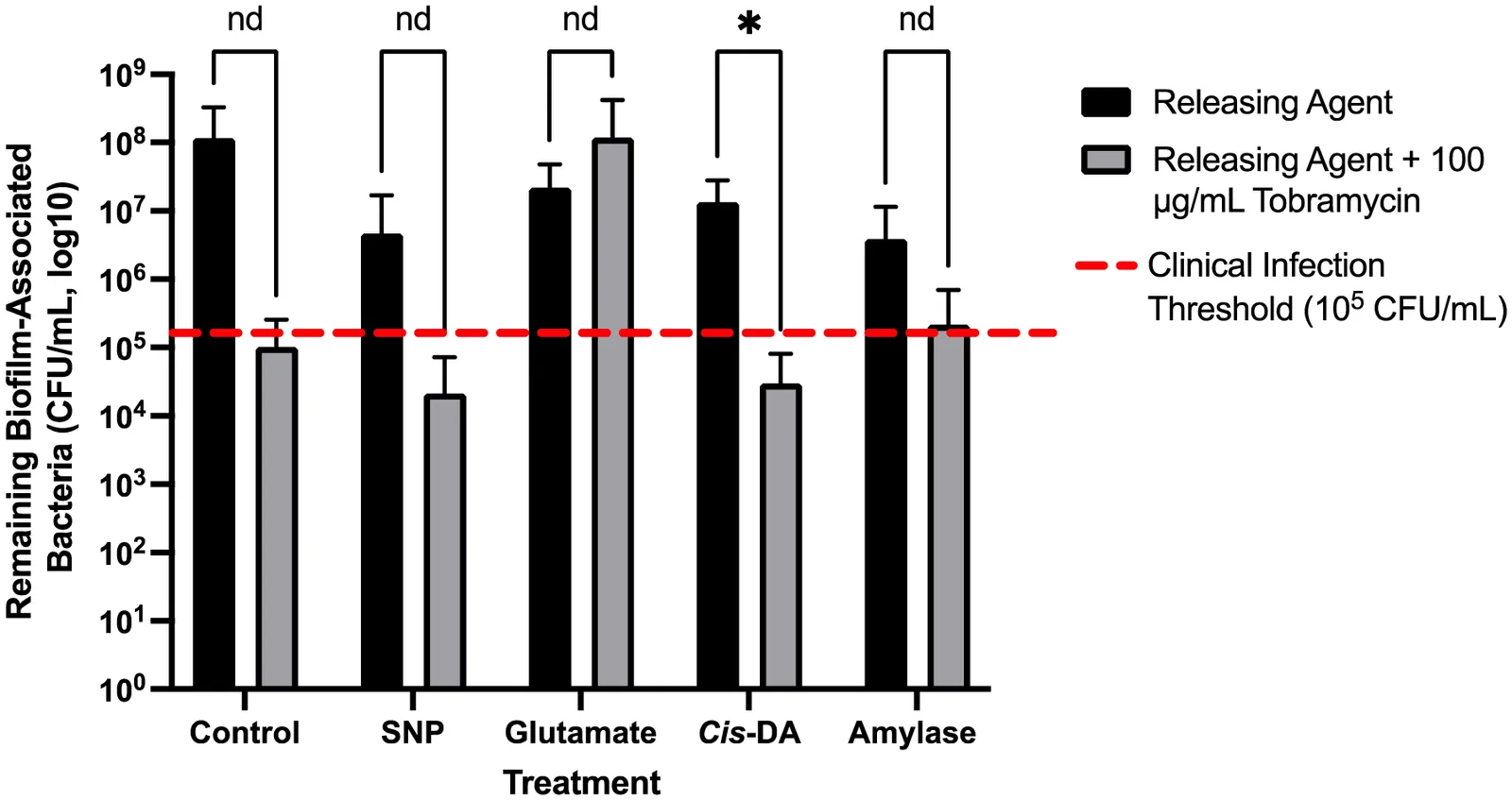
Tobramycin-mediated killing of 4-day *P. aeruginosa* biofilms following treatment with releasing agents. 4-day PAO1 biofilms were treated for 2 h with LB (control), 100 µg/mL tobramycin alone, or each releasing agent with or without 100 µg/mL tobramycin. Following treatment, the remaining biofilm biomass was rinsed, scraped from the wells, homogenized, serially diluted, and drop-plated to enumerate CFU/mL Statistical significance was determined using two-way ANOVA followed by Sidak's multiple comparisons test comparing treatments in the absence and presence of tobramycin. “nd” indicates no significant difference. The red-dashed line indicates a clinical infection threshold (10^5^ CFU/mL). Mean ± standard deviation is shown (*p < 0.05). n = 7–18/group.

## 4. Conclusions

Several researchers have directly compared selected biofilm dispersion mechanisms and demonstrated that the mode of dispersion can influence the physiology of released cells [19],[20],[31]–[34]. However, these researchers have primarily examined signaling-mediated and physical mechanisms of dispersion. In this study, we extend these comparisons by evaluating four mechanistically distinct release approaches, including enzymatic matrix degradation by amylase across phenotypic and transcriptional outcomes. This side-by-side comparison demonstrates that the mechanism used to release biofilm cells substantially influences the resulting phenotypic and transcriptional characteristics.

A key observation from this study is that exposure of planktonic cells to the releasing agents alone produced only modest changes in phosphodiesterase (PDE) activity (Figure 1B). Because PDE activity was assessed using a synthetic substrate rather than by direct quantification of intracellular c-di-GMP, these results should be interpreted as differences in PDE activity associated with c-di-GMP metabolism rather than direct evidence of altered intracellular c-di-GMP concentrations. Although transient reductions in growth were observed following exposure to *cis*-DA and SNP, these effects were resolved within the experimental timeframe (Figure 1A), and downstream phenotypic analyses were performed outside this transient growth inhibition period. This transient inhibition aligns with other observations that NO donors can modulate cell cycle progression and temporarily reduce metabolic activity without inducing permanent stress responses [35],[36]. Together, these findings indicate that the physiological differences observed among released-cell populations are unlikely to result solely from direct exposure to the releasing agents, but instead are associated with the biofilm release process.

The release mechanisms examined in this study generated distinct populations of released cells, although no statistically significant differences in the extent of biofilm release were detected among the four treatments (Figure 2). Consistent with other reports, *cis*-DA and SNP primarily released individual bacterial cells, whereas amylase-mediated degradation of the extracellular polymeric substance (EPS) resulted in the release of multicellular aggregates that remained partially associated with the biofilm matrix (Figure S2) [20],[33],[34].

The ability of SNP and glutamate to induce biofilm dispersion is consistent with other studies demonstrating dispersion in response to SNP and nutrient cues. Moreover, comparative studies have demonstrated that the mechanism used to induce dispersion can influence the characteristics of released *P. aeruginosa* cells, including c-di-GMP levels, antibiotic susceptibility, and other physiological traits [18],[21],[24],[25],[37],[38].

Although glutamate also released a large proportion of biofilm-associated cells, these released populations differed phenotypically from those generated by the other release mechanisms. Although not statistically significant, glutamate-released cells exhibited greater survival following tobramycin exposure than the other released-cell populations (Figure 3). Glutamate and amylase released cells exhibited significantly greater reversible attachment than SNP and *cis*-DA released cells (Figure 4), demonstrating that the mechanism of biofilm release can influence the subsequent attachment behavior of released cells.

Our findings are broadly consistent with evidence that the physiological properties of released cells depend on the mechanism of release. Wille et al. [32] directly compared actively dispersed cells generated by glutamate and SNP with spontaneously and passively dispersed populations and found that the dispersion trigger influenced antibiotic susceptibility with glutamate-released cells exhibiting reduced susceptibility to colistin compared with SNP-released cells. In this study, glutamate-released cells similarly exhibited less susceptibility to tobramycin, although differences among the released-cell treatment groups did not reach statistical significance. Together, these observations suggest that nutrient-induced and SNP-mediated release may produce populations with different antimicrobial susceptibility.

Preliminary gene expression analyses suggest that released-cell populations differed according to the mechanism of release (Figure 5). The largest transcriptional differences were observed for genes associated with biofilm regulation (*bdlA*), antibiotic resistance (*mexR*), and to a lesser extent, quorum sensing (*lasR*), whereas relatively few differences were detected among motility-associated genes. Glutamate and amylase-released cells generally exhibited the greatest transcriptional differences relative to the other release mechanisms, while SNP and *cis*-DA-released cells displayed expression patterns that were more similar across the genes examined. Although we evaluated a targeted panel of regulatory genes rather than the entire transcriptome, these findings suggest that different release mechanisms generate transcriptionally distinct released-cell populations.

Differences between release mechanisms were also evident in the biofilm recovery and biofilm reformation assays (Figure 6). Recovery of the residual biofilm following treatment and biofilm formation by released cells varied throughout the 72-h observation period, indicating that the release mechanism influenced the remaining biofilm and the subsequent biofilm-forming capacity of released cells. These findings are consistent with previous studies suggesting that biofilm dispersal is not a uniform process and that the physiological characteristics of released cells can vary depending on how biofilm release is induced [21],[31]. Here, we extend these observations by incorporating enzymatic matrix degradation alongside signaling-mediated release mechanisms and evaluating multiple downstream characteristics of the resulting released-cell populations.

The influence of release mechanisms on antibiotic efficacy represents an important consideration for the development of biofilm-directed therapies. Consistent with the susceptibility assays performed on released cells (Figure 3), glutamate-treated biofilms exhibited little improvement following tobramycin treatment, whereas *cis*-DA treatment significantly enhanced antibiotic-mediated killing of the remaining biofilm (Figure 7). Although SNP and amylase-treated biofilms also exhibited lower bacterial burdens following treatment, these reductions were not statistically significant and therefore should be interpreted cautiously. Nevertheless, both treatments reduced the remaining bacterial burden below the clinical infection threshold of 10^5^ CFU/mL, suggesting that further investigation of these combinations may be warranted. Collectively, these findings indicate that combining biofilm release with antimicrobial therapy does not produce equivalent outcomes across release mechanisms and that the method used to induce biofilm release should be considered when developing dispersion-based treatment strategies.

In summary, this study demonstrates that different biofilm release mechanisms generate released-cell populations with distinct phenotypic and transcriptional characteristics. Direct comparison of 4 mechanistically distinct release strategies revealed differences in antibiotic susceptibility, reversible attachment, gene expression, biofilm recovery, and biofilm reformation, despite comparable biofilm release efficiency. These findings emphasize that biofilm release is not a uniform process and suggest that the mechanism used to induce biofilm release should be carefully considered when evaluating dispersion-based therapeutic approaches. Future studies incorporating global transcriptomic analyses, direct measurement of intracellular signaling molecules, and clinically relevant infection models will further define the biological and therapeutic significance of these distinct released-cell populations.

## 5. The limitations of this study

This study has several limitations that should be considered when interpreting the findings. First, the experiments were performed using an *in vitro* biofilm model and therefore did not account for host immune responses or other factors present during infection. An additional limitation of the *in vitro* biofilm models was the use of two biofilm maturation periods across experimental workflows. Moreover, 2-day biofilms were used for released-cell characterization because they provided sufficient recoverable released-cell populations for downstream phenotypic and molecular analyses, whereas mature 4-day biofilms were used to evaluate biofilm release and antibiotic-mediated killing. Although biofilm age was held constant across treatment groups within each experimental workflow, differences in biofilm maturity may influence matrix composition, cellular physiology, and responses to releasing agents. Therefore, direct quantitative comparisons between experiments performed using 2- and 4-day models should be interpreted cautiously. Future studies directly comparing release cells from biofilms of different maturities under otherwise identical conditions will be important for determining the extent to which biofilm age influences released-cell phenotypes and transcriptional profiles.

Second, gene expression analyses were limited to a targeted panel of seven genes associated with motility, biofilm formation, dispersion, antibiotic resistance, quorum sensing, and chronic infection. Therefore, our findings represent expression patterns of selected genes rather than a comprehensive transcriptional profile. Broader transcriptomic analyses, such as RNA-seq, will be necessary to fully characterize the transcriptional responses associated with different releasing mechanisms. While these genes represent important regulators of biofilm physiology, broader transcriptomic or proteomic analyses will be necessary to fully characterize the molecular responses associated with different release mechanisms. Additionally, the RT-qPCR analyses were performed with a limited number of biological replicates (n = 2–3), which limits statistical power and increases sensitivity to observations. Although technical duplicates and multiplicity-adjusted statistical analyses were used, the transcriptional findings should be considered preliminary and require validation with additional biological replicates. Additionally, gene expression was normalized using a single reference gene (*rho*), and the stability of *rho* was not independently validated across the populations. Moreover, reference-gene expression in *P. aeruginosa* can vary according to physiological and environmental conditions and biofilm heterogeneity can further complicate the identification of universally stable housekeeping genes [39],[40]. Therefore, researchers should validate multiple candidate reference genes across the specific released conditions and use multiple validated reference genes for normalization.

Finally, intracellular c-di-GMP concentrations were not measured directly. Although the bis-pNPP PDE assay is an established approach for assessing phosphodiesterase activity associated with c-di-GMP metabolism, PDE activity does not directly quantify intracellular c-di-GMP concentrations. Future studies incorporating direct c-di-GMP quantification, such as LC-MS/MS or c-di-GMP responsive reporter systems, will be necessary to confirm the relationship between the observed PDE activity and intracellular c-di-GMP levels.

## Use of AI tools declaration

The authors declare they have not used Artificial Intelligence (AI) tools in the creation of this article.


