# Supplementary material for: Uncovering the phenotypic and transcriptional characteristics of biofilm-released cells in Pseudomonas aeruginosa
Source: AIMS Microbiol. 2026 Sep 30;12(3):718–41. doi: 10.3934/microbiol.2026031 (PMC13652744; doi:10.3934/microbiol.2026031)
Supplement: Supplementary file 1 [file microbiol-12-03-031-s001.pdf]

Microbiology

AIMS Microbiology, 12(3): 718–741.
DOI: 10.3934/microbiol.2026031
Received: 12 July 2026
Revised: 18 September 2026
Accepted: 29 September 2026
Published: 30 September 2026

https://www.aimspress.com/journal/microbiology

Research article

Uncovering the phenotypic and transcriptional characteristics of biofilm-released cells in *Pseudomonas aeruginosa*

Jason Li[1,†], Larry Ayiku[1,2,†], Benjamin Larson[1,2,†], Agam Patel[1,2], Dylan Adams[1,2], Patrick Moran[1], Samantha Burgan[1,2], Esha Shah[1], Anthony Bruno[1,2], Jaclyn Printz[1,2], Tevin Flom[1,2] and Whitni K. Redman[2,*]

[1] Department of Biological Sciences, Binghamton University, Binghamton, NY, United States
[2] First-year Research Immersion Program, Binghamton University, Binghamton, NY, United States

* **Correspondence:** Email: wredman@binghamton.edu.

† These three authors contributed equally.

Supplementary

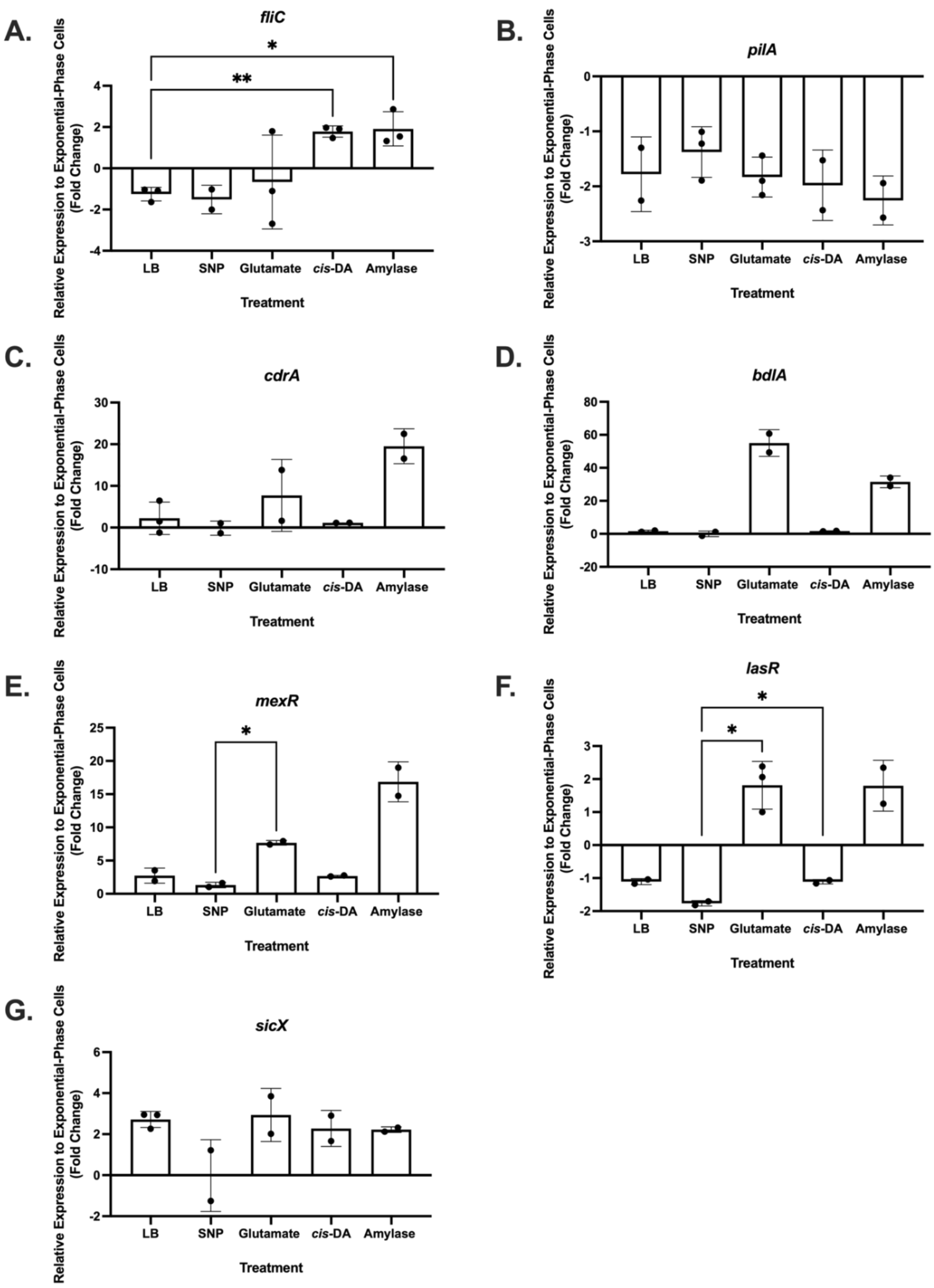

Figure S1. Gene expression of biofilm-released *Pseudomonas aeruginosa* cells normalized to exponential-phase controls. RT-qPCR analysis of gene expression in PAO1 cells released from 2-day biofilms following treatment with LB (control), SNP, glutamate, *cis*-DA, or amylase. Released cells were collected, RNA was extracted, and relative gene expression was calculated using the ΔΔCt method normalized to untreated exponential-phase cells. Panels represent relative gene expression for (A) *fliC*, (B) *pilA*, (C) *cdrA*, (D) *bdlA*, (E) *mexR*, (F) *lasR*, and (G) *sicX*. Statistical significance was determined using Brown-Forsythe and Welch ANOVA followed by Games-Howell multiple-comparisons testing with multiplicity-adjusted P values. Mean ± standard deviation is shown (*$p < 0.05$; **$p < 0.01$). n = 2–3 independent biological replicates/group, with each biological replicate analyzed in technical duplicate and technical duplicate values averaged prior to statistical analysis.

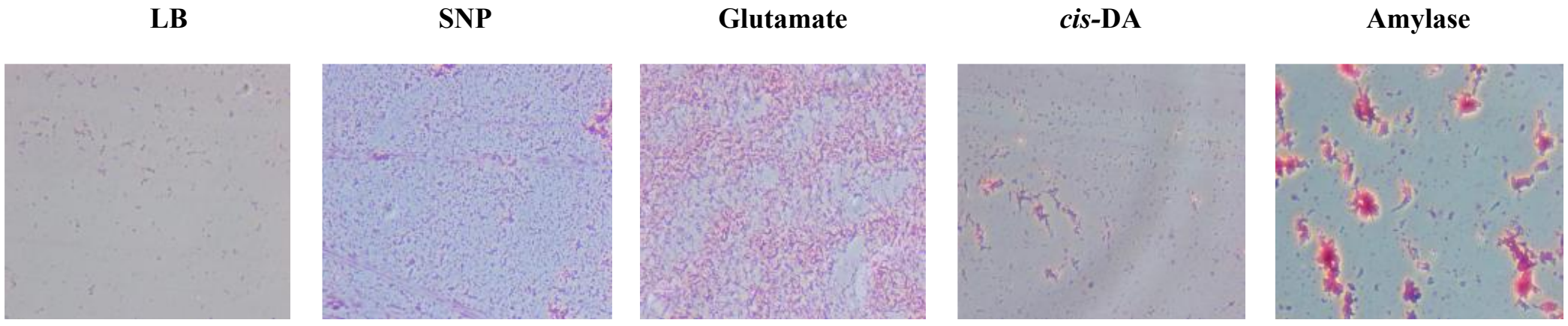

Figure S2. Microscopy of released-cell populations following treatment with various releasing agents shows distinct morphological differences. The micrographs above show the resulting cell populations in the bulk supernatant after treatment with 1 mL of full-strength LB, 500 µM SNP, 18 mM glutamate, 310 nM *cis*-DA, or 500 U/mL amylase. The LB-treated sample shows a small abundance of free cells in the liquid. Conversely, SNP and glutamate treated samples show a high abundance of individual free cells with minimal matrix-bound aggregates of biofilm cells. *cis*-DA treatment overwhelmingly results in free cells with a few aggregates and visible lysed cells. Amylase shows the lowest abundance of free cells with cells being arranged in matrix-bound aggregates of cells.

Table S1. RT-PCR primers.

Gene	Function	Forward Primer (5'-3')	Reverse Primer (5'-3')
rho	Transcription termination factor	ATGAAGATGGAAGCCGGCAA	TGCCGGTACCCTTGAATTCC
fliC	Motility and attachment	CGAACTGACCCGTATCTCCG	TCGTTGATGCTGGCAGTGAT
pilA	Motility and attachment	ACTGATGATCGTGGTTGCGAT	TCGGAGTGAACATCGGATCC
cdrA	Biofilm formation	GCCCAGGTGGAGATGTTCAA	AGCTGAGGGTGTAATTGGCC
sicX	Acute to chronic infections	GAGTGGAACGTGCGGAGAG	GTTGCTCGTCGTTTTCCACC
bdlA	c-di-GMP levels and dispersion	ACCAGGAACACGAGATGCAG	TGACAGTTCGCCGATGTTCA
lasR	c-di-GMP levels and dispersion	GGCCTTGGTTGACGGTTTTC	TTGCTGACCGGATGTTCGAA
mexR	Drug resistance	GAACTACCCCGTGAATCCCG	TTGCTGACCGGATGTTCGAA

AIMS Press

© 2026 the Author(s), licensee AIMS Press. This is an open access article distributed under the terms of the Creative Commons Attribution License (https://creativecommons.org/licenses/by/4.0)
